# The Hong Kong Grocery Shopping Dialog Task (HK-GSDT): A Quick Screening Test for Neurocognitive Disorders

**DOI:** 10.3390/ijerph192013302

**Published:** 2022-10-15

**Authors:** Xianmin Gong, Patrick C. M. Wong, Helene H. Fung, Vincent C. T. Mok, Timothy C. Y. Kwok, Jean Woo, Ka Ho Wong, Helen Meng

**Affiliations:** 1 Stanley Ho Big Data Decision Analytics Research Centre, The Chinese University of Hong Kong, Hong Kong, China; 2 Department of Psychology, The Chinese University of Hong Kong, Hong Kong, China; 3 Department of Linguistics and Modern Languages, The Chinese University of Hong Kong, Hong Kong, China; 4 Brain and Mind Institute, The Chinese University of Hong Kong, Hong Kong, China; 5 Department of Otorhinolaryngology, Head and Neck Surgery, The Chinese University of Hong Kong, Hong Kong, China; 6 Division of Neurology, Department of Medicine and Therapeutics, The Chinese University of Hong Kong, Hong Kong, China; 7 Gerald Choa Neuroscience Centre, Margaret K. L. Cheung Research Centre for Management of Parkinsonism, Therese Pei Fong Chow Research Centre for Prevention of Dementia, Lui Che Woo Institute of Innovative Medicine, The Chinese University of Hong Kong, Hong Kong, China; 8 Li Ka Shing Institute of Health Sciences, The Chinese University of Hong Kong, Hong Kong, China; 9 Department of Medicine and Therapeutics, The Chinese University of Hong Kong, Hong Kong, China; 10 Jockey Club Centre for Osteoporosis Care and Control, The Chinese University of Hong Kong, Hong Kong, China; 11 Jockey Club Institute of Aging, The Chinese University of Hong Kong, Hong Kong, China; 12 Department of Systems Engineering and Engineering Management, The Chinese University of Hong Kong, Hong Kong, China

**Keywords:** neurocognitive disorder, dementia, mild cognitive impairment, screening, early detection, neurocognitive test, instrumental activity of daily living

## Abstract

The Hong Kong Grocery Shopping Dialog Task (HK-GSDT) is a short and easy-to-administer cognitive test developed for quickly screening neurocognitive disorders (NCDs). In the test, participants are instructed to do a hypothetical instrumental activity of daily living task of purchasing ingredients for a dish from a grocery store and verbally describe the specific shopping procedures. The current study aimed to validate the test with a sample of 545 Hong Kong older adults (58.8% female; aged 73.4 ± 8.37 years), including 464 adults with normal cognitive function, 39 with mild NCD, and 42 with major NCD. Demographic characteristics (i.e., sex, age, education) and clinical diagnosis of cognitive states (i.e., major NCD, mild NCD, and normal aging) were collected. Cognitive functioning was measured using the HK-GSDT and several standardized NCD-screening tests. The results showed good reliability (i.e., internal consistency) and structural validity in the HK-GSDT. It discriminated among different cognitive conditions, particularly between major NCDs and the other conditions, as effectively as did the existing standardized neurocognitive tests (e.g., Montreal Cognitive Assessment, Hong Kong List Learning Test). Moreover, the HK-GSDT explained additional variance of cognitive condition on top of those standardized neurocognitive tests. These results indicate that the HK-GSDT can be used alone, or in combination with other tests, to screen for NCDs.

## 1. Introduction

Neurocognitive disorder (NCD) is an umbrella term referring to cognitive deterioration and impairments worse than normative cognitive declines from human development and aging, as per the fifth edition of the Diagnostic and Statistical Manual of Mental Disorders (DSM-5) [1]. Based on their etiologies, NCDs are classified into different types, primarily including NCDs caused by Alzheimer’s disease (AD), vascular dementia (VD), Lewy body dementia (LBD), Parkinsonian dementia (PD), and frontotemporal dementia (FTD). According to the severity of symptoms, NCDs are divided into two stages: mild NCDs (m-NCDs, the earlier stage of NCDs; similar to mild cognitive impairment) and major NCDs (M-NCDs, the later stage of NCDs; also known as dementia). NCDs are prevalent worldwide: There are more than 55 million people with dementia (M-NCDs), and this number grows by nearly 10 million every year [2]. It is crucial and imperative to effectively screen and diagnose NCDs timely. For this purpose, we have developed the Hong Kong Grocery Shopping Dialog Task (HK-GSDT), a quick (about 10-min), easy-to-administer, and valid test that can be used alone, or together with other tests, for screening for NCDs. In this paper, we will explain the motivation for developing the HK-GSDT and validate it.

### 1.1. Cognitive Deficits Associated with Different Types of NCDs

**Figure 1:**
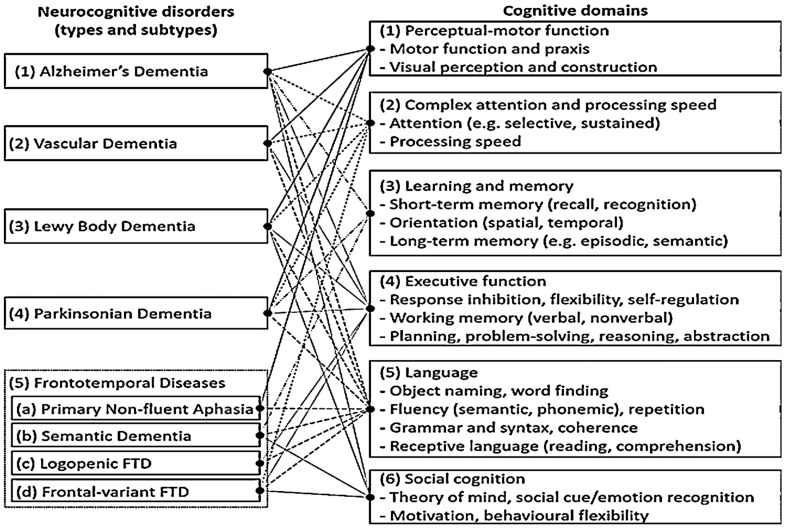
Different types of neurocognitive disorders and associated deficits in various cognitive domains. Links to different cognitive domains are distinguished by different types of lines. The figure is adapted from Lay et al. (under review) [3].

As summarized in Figure 1 [3], different types of NCDs are characterized by deficits in six key cognitive domains (also see DSM-5 [1]): (1) NCDs due to Alzheimer’s disease (AD) primarily cause deficits in learning and memory, followed by deficits in the domains of executive function and perceptual-motor function [4,5]; (2) NCDs due to vascular dementia (VD) mainly impact the domains of executive function, motor function, and attention and processing speed [6,7]; (3) NCDs due to Lewy body dementia (LBD) impair attention and processing speed, perceptual function and executive function [5,8]; (4) NCDs due to Parkinsonian dementia (PD) are primarily associated with deficits in motor function, followed by deterioration in the domains of attention, executive function and visuospatial abilities [4,9]; (5) Three variants of NCDs caused by frontotemporal diseases (FTD)—primary non-fluent aphasia, semantic dementia, logopenic FTD—are primarily associated with language impairments, and the fourth variant (i.e., frontal-variant FTD) are mainly associated with changes in social cognition and behavior [7,10,11]. Noteworthy, despite the specificity of cognitive deficits associated with different types of NCDs (especially in the early stages), they all tend to cause deficits across most cognitive domains as they progress (as shown in Figure 1).

### 1.2. Neurocognitive Tests for Screening NCDs

As described above, different types of NCDs tend to show different cognitive symptoms. Hence, an appropriate screening test (or test battery) should cover a wide range of relevant cognitive functions while remaining brief enough so that it can be used to detect NCDs of various causes [12]. Indeed, the literature has shown that comprehensive tests (i.e., tests that cover a wide range of cognitive domains) are more sensitive to detecting NCDs than tests with restricted coverage of cognitive domains [12,13,14].

A large number of neurocognitive tests have been developed to screen or detect NCDs [12,13,14,15,16]. Only a handful of comprehensive tests—such as the Addenbrooke’s Cognitive Examination Revised (ACE-R) [17], the Montreal Cognitive Assessment (MoCA) [18], and the Short Test of Mental Status (STMS) [19]—cover most key cognitive domains [12,14], except for social cognition, a recently recognized cognitive domain that is neglected by most screening tools [3,6]. However, no single test to date performs well enough to detect NCDs of various causes in different contexts [12,13,20], which implies room for improvement. One of the issues is that these tests miss some crucial cognitive functions, such as non-verbal episodic memory (e.g., spatial memory) and dialog speech.

The HK-GSDT is designed by us to fill in this gap and contribute to improving the effectiveness of NCD screening. It is a comprehensive yet brief screening test for NCDs, which can be used alone or in combination with other tests. In the following sections, we will introduce the task, discuss its strengths (e.g., brevity, low test bias, a wide range of cognitive domains, and the inclusion of several key cognitive functions that are not covered by existing screening tools), and then test its psychometric quality and effectiveness in detecting NCDs.

### 1.3. The HK-GSDT

The HK-GSDT is a 10 min structural cognitive test. During the entry section (about 2 min), a tester shows the participant pictures of a grocery store and a dish (see Figure S1 in the Supplementary Materials) and explains the hypothetical shopping task to the participant. During the main task section (about 6 min), under the tester’s guidance, the participant needs to name the ingredients for the dish, describe the steps of cooking the dish, and narrate the specific processes of purchasing the ingredients (including how to reach the target aisles and how to select the desired ingredients). The main task section is followed by a memory retrieval task (about 2 min), in which the participant needs to recall which aisle s/he was last in, as well as the location of a specified product (e.g., pineapple bun) that is not one of the gradients. The following are two examples of dialog between the tester and participants during the test:

Dialog example 1—

Tester: Which ingredients do you see in this soup photo?

Participant: I see tofu, pork, egg, and tomato.

Dialog example 2—

Tester: In case you want to cook the soup, what ingredient you would buy first from the store?

Participant: I would buy pork first.

Tester: How would you walk from the entrance to the shelf to get pork?

Participant: I would go straight to the end of the aisle, turn left, and then turn right to get pork from the “Meat” shelf.

The participant’s performance is scored on five items: (1) the number of ingredients s/he has correctly figured out (one point for each correct ingredient; with the maximum score on this item being four points); (2) the number of aisles (where the ingredients are) s/he has correctly identified (one point for each correct aisle; with the maximum score on this item being four points); (3) the number of aisles s/he has wrongly identified (−1 point for each wrong aisle; with the possible score on this item ranging between −4 and 0); (4) whether s/he has correctly recalled her/his final location (one point for correct and zero point for incorrect recall); and (5) whether s/he has correctly recalled the location of a given product (one point for correct and zero point for incorrect recall). The total score of the whole test (i.e., the summed score across the five items) ranges from 0 to 10, with a higher score indicating better cognitive functioning.

### 1.4. Strength of the HK-GSDT

An appropriate NCD screening tool should be simple, easy to administer, and acceptable to the participants being tested. It should also be robust against biasing factors unrelated to NCDs, such as demographics, as well as be able to detect NCDs of various causes effectively [12,13,14]. The HK-GSDT meets these requirements well.

#### 1.4.1. Brevity and Ease of Administration

The HK-GSDT is a brief, structural test that should take approximately 10 min only. The test can be administered and scored by non-specialists with minimal training. The task is also acceptable to the participants, as it involves a hypothetical daily shopping chore that almost every adult has to perform in their daily lives.

#### 1.4.2. Promisingly Low Test Bias

The performance of participants in NCD screening tests is often biased by factors unrelated to NCDs, such as demographics (e.g., age, sex, and education) and contextual factors (e.g., the familiarity of the test to the participants) [12,13,21]. Studies have shown that traditional neurocognitive tests in laboratory settings, due to relatively low ecological validity (e.g., low familiarity), may underestimate the cognitive capacities of older adults. Older adults often display deficits in laboratory-based neurocognitive tests but relatively well-preserved cognitive competence in everyday life situations [22,23]. The HK-GSDT shows promise to mitigate such bias due to its high ecological validity—It captures everyday cognitive competence by modeling a common instrumental activity of daily living, namely shopping, that is familiar to almost everyone, and thus the participants’ performance should be minimally affected due to such unfamiliarity. Tests based on instrumental activities of daily living, such as Communication Activities of Daily Living (CADL) [24], have been frequently used to measure cognitive functions in older adult, and such tests often perform better than laboratory tests [22,23]. The HK-GSDT also requires no knowledge acquired via formal education and thus should have low educational bias.

#### 1.4.3. Wide Coverage of Key Cognitive Functions

As summarized in Table 1, the HK-GSDT covers all key cognitive domains discussed above (except for social cognition). Although the test does not provide individual scores for each cognitive function, a good performance on the test depends on well-preserved functions across all these domains. Deficits in any of these domains could lower performance on the test. Thus, the test should be able to detect different types of NCDs, which are associated with different cognitive deficits.

#### 1.4.4. Coverage of Cognitive Functions Neglected in the Other Screening Tests

The HK-GSDT covers several cognitive functions that are seldomly covered in other tests for screening NCDs, including dialog function, the function of naming actions, and non-verbal episodic memory.

**(1) Dialog function.** Good performance on the HK-GSDT requires intact dialog functions (as participants are required to converse with testers as part of the task), which is an essential aspect of language function. Language deficits are primary symptoms of NCD due to FTD, and they also often appear in other types of NCDs, such as those caused by AD, VD, PD, and LBD [4,7,10]. In previous NCD screening tests, language deficits are typically detected at the word-, verbal-, or sentence levels (e.g., naming of objects, verbal fluency, sentence repetition) [12,13,14]. Only a small number of tests measure connected speech functions using monolog tasks, such as picture-describing tasks and story-telling tasks [25]. To our knowledge, no established NCD screening test examines dialog function. A large body of evidence demonstrates that dialog speeches are very useful in detecting different types of NCDs [26,27,28,29], which are associated with different language deficits at different levels ranging from phonetic and articulatory features [30] to discourse [31] and pragmatic features [3,32,33]. Compared with those commonly used simple language tasks (e.g., naming tasks, sentence repetition tasks), dialog tasks are more capable of capturing diverse language features and deficits [4,34].

**(2) Non-verbal episodic memory.** The HK-GSDT covers semantic memory/knowledge, verbal episodic memory, and non-verbal episodic memory (see Table 1). Memory problems are one of the most common symptoms of NCDs and are examined in most NCD screening tests [12,13,14], although DSM-5 [1] excludes impairments in learning and memory from the diagnostic criteria for M-NCDs [35]. In NCD screening tests, semantic memory and short-term verbal episodic memory, such as memory of the names of objects, are typically assessed because these forms of memory are typically impaired in NCDs, whereas some other forms of memory, such as implicit memory and autobiographic memory, often remain relatively intact [4,36]. However, numerous studies have shown that NCDs can lead to problems in non-verbal episodic memory (e.g., visuospatial memory, topographical memory, associative memory, and source memory), especially when there is hippocampal damage [37,38,39,40]. For example, it has been found that the Paired Associate Learning Test, in which participants learn and memorize the association between spatial locations and colored patterns, is highly sensitive for detecting early AD [41,42]. In addition, impairments in semantic and verbal episodic memory may be confounded by language deficits (since the tests of such memory are language-based), whereas non-verbal episodic memory suffers less from such effects [39]. Therefore, the inclusion of examinations on non-verbal episodic memory tests can contribute to NCD screening.

**(3) Naming of actions.** In the HK-GSDT, participants need to name not only objects (e.g., the ingredients of a dish) but also actions (e.g., specific actions involved in cooking and shopping). In NCD screening tests, the naming function is often examined as part of memory and language functions, and these tests typically focus on object naming while overlooking action naming [12,13]. Different types of NCDs may be associated with different naming difficulties: object-naming difficulties are more common in NCDs caused by AD, VD, LBD, and FTD, while action-naming difficulties are more common in NCDs caused by PD [4,10,43]. The coverage of action-naming functions may be particularly helpful for the screening of NCDs due to PD.

### 1.5. Current Study

In this study, we aim to examine the effectiveness of the HK-GSDT for NCD screening using a Hong Kong older adult sample. We expected the test to display good psychometric properties (namely good reliability, good validity, and low levels of test bias). Moreover, we anticipated that this test, both as a stand-alone test and a test to be combined with other tests, can accurately distinguish between older adults with different cognitive statuses (e.g., normal aging, m-NCD, and M-NCD) regardless of NCD type.

## 2. Materials and Methods

### 2.1. Sample

Data were collected from 545 older Hong Kong adults. As shown in Table 2, participants were classified into three cognitive health conditions (i.e., M-NCD, m-NCD, and normal aging) based on their neurocognitive test scores and two clinicians’ diagnoses. Two participants in the M-NCD condition and 12 participants in the normal-aging condition had missing values on some measures. Their data were deleted using the pairwise deletion method. The research ethics approval was obtained from the medical ethics committee of The Chinese University of Hong Kong and New Territories East Cluster of Hospital Authority of Hong Kong.

### 2.2. Measures

In addition to the HK-GSDT, several well-established cognitive tests were also administered to the participants to assess the convergent and divergent validity of the HK-GSDT. The tests used for convergent validation included the 5 min version and the full version of the Montreal Cognitive Assessment [18] Hong Kong Version (HK-MoCA, a comprehensive cognitive test) [44], Hong Kong List Learning Test (HKLLT) 2nd Edition (a test for verbal learning, verbal memory and use of organizational strategies) [45], Wechsler’s backward/forward digit span tasks and logical memory recognition task (tests for working memory and verbal episodic memory) [46], and the modified Boston Naming Test with 30 items (mBNT, a test for perceptual and language abilities) [47,48]. Note that the widely used Mini-Mental State Exam (MMSE) [49] was not included, as most of the tasks in MMSE are covered by MoCA. Given that these tests overlap with the HK-GSDT in terms of the coverage of cognitive functions, we expected participants’ scores on these tests to be highly correlated with their scores on the HK-GSDT. The tests used for divergent validation included the Eight-item Interview to Differentiate Aging and Dementia (AD8, a test for self-perception of cognitive abilities and daily function) [50] and the Color Trails Test (CTT, a test for visual tracking and motor execution abilities) [51]. Another test for divergent validation was the five-item version of the Geriatric Depression Scale (GDS) measuring depressive symptoms [52]. Depressive symptoms often contribute to cognitive declines but are not very relevant to NCDs [35]. These tests measure different constructs than the HK-GSDT, and we expected participants’ scores on these tests to have low correlations with scores on the HK-GSDT. Demographic variables (age, gender, and education) were used to assess the test bias of the HK-GSDT and were treated as covariates in the regression analyses.

### 2.3. Analytical Strategies

To validate the HK-GSDT, internal consistency was calculated to assess its measurement reliability. Then, several types of validity (construct validity, divergent and convergent validity, and criterion validity) and test bias of the test were assessed. As a final step, we examined whether the HK-GSDT could be applied together with other tests to improve the accuracy of NCD screening.

## 3. Results

### 3.1. Reliability and Structural Validity

As described in the introduction, participants’ performance on the HK-GSDT was scored using five items, including the number of correctly identified ingredients, number of correctly identified aisles, number of wrongly identified aisles, correct recall of the final location, and correct recall of a specified product. Cronbach’s α of these five items was 0.70, and McDonald’s ω was 0.79, indicating acceptable internal reliability of the test.

Exploratory factor analysis was conducted to evaluate the structural validity of the HK-GSDT. The result showed that the five items loaded on one latent factor, namely cognitive function (see Figure S2 in the Supplementary Materials). The latent factor accounted for 47.1% of the total variance in the five items.

### 3.2. Divergent and Convergent Validity

The correlations between the HK-GSDT scores and scores on the other measures are displayed in Table 3. As we expected, there were medium to strong correlations between the HK-GSDT scores and the scores on the other tests measuring similar cognitive functions (e.g., memory, language, executive function, perceptual ability), including the logical memory test, digit span tests, HKLLT, mBNT, and MoCA. The results indicated good convergent validity of the HK-GSDT. The HK-GSDT scores have low correlations with the demographic variables (i.e., age, gender, and education), and other tests (i.e., CCT, AD8, and GDS) gauging dissimilar constructs (e.g., visual tracking and motor execution, self-perception of daily function, depression), indicating good divergent validity of the HK-SDT.

### 3.3. Criterion Validity: Effectiveness of the HK-GSDT in NCD Screening as a Standalone Test

**Figure 2:**
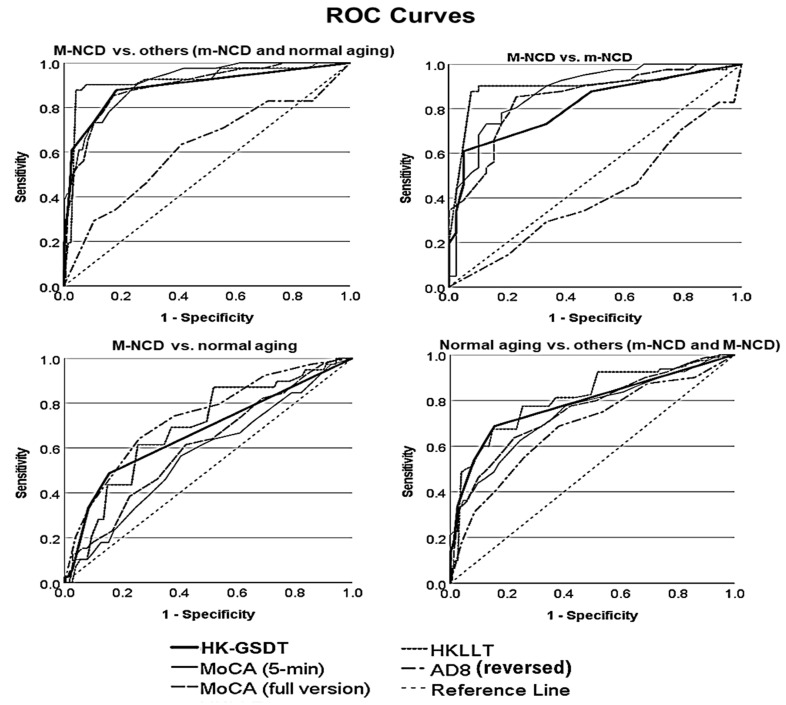
Receiver operating characteristic (ROC) curves for different tests to discriminate between cognitive conditions. A curve closer to the top-left corner indicates better performance in discriminating between cognitive conditions. HK-GSDT = Hong Kong Grocery Shopping Dialog Test; MoCA = Montreal Cognitive Assessment Hong Kong Version; HKLLT = Hong Kong List Learning Test (2nd Edition); AD8 = Eight-item Interview to Differentiate Aging and Dementia (the scores on AD8 were reversed coded, such that a higher score indicated poorer self-perceived cognitive functions).

Receiver operating characteristic (ROC) analyses were conducted to examine how well each cognitive test discriminated between different cognitive health conditions. Figure 2 displays the ROC curves, and Table S1 in the Supplementary Materials lists the areas under these curves. The results showed that the HK-GSDT performed as well as MoCA and HKLLT (and they all performed better than AD8) in discriminating between M-NCD and the other two cognitive conditions (i.e., m-NCD and normal aging), or discriminating between M-NCD and m-NCD. However, none of these tests alone could discriminate between normal aging and m-NCD well. Overall, the results suggest that the HK-GSDT performs similarly well as other established neurocognitive tests (e.g., MoCA, HKLLT) in screening for NCDs.

### 3.4. Criterion Validity: Effectiveness of the HK-GSDT in NCD Screening in Combination with Other Tests

Multinomial logistic regression analyses were performed to examine whether the HK-GSDT explained additional variability in cognitive status (i.e., normal aging, m-NCD, or M-NCD) on top of other established neurocognitive tests. Demographics (age, gender, and education years) were controlled as covariates. As shown in Table S2 in the Supplementary Materials, HK-GSDT increased the model fit on top of MoCA (5-min), MoCA (full version), HKLLT, and AD8, respectively (i.e., AIC, BIC, −2 log-likelihood generally became smaller, and model comparison showed significant model improvement), and the amount of explained variance (pseudo-R2) increased by 3%–12%. The results suggest that the HK-GSDT could be used in conjunction with other tests to improve NCD screening accuracy.

### 3.5. Test Bias Related to Demographics

As shown in Table 3, participants’ scores on the HK-GSDT were not correlated with gender, weakly correlated (r = 0.12) with education, and moderately correlated (r = −0.42) with age. The results suggest that performance on the test may be biased by age but not by gender or education, and we may need to develop different norms and screening criteria based on the HK-GSDT for people of different ages. We then examined whether the HK-GSDT predicted cognitive status (i.e., normal aging, m-NCD, M-NCD) equally well for different genders, ages, and education levels. Multinomial logistic regression analyses showed no significant HK-GSDT × education interaction effect in predicting cognitive status (*p* = 0.39), with age and gender controlled for; no significant HK-GSDT × gender interaction (*p* = 0.64), with age and education controlled for; and no significant HK-GSDT × age interaction (*p* = 0.07), with gender and education controlled for. The results suggest that the HK-GSDT is similarly effective in screening for NCDs at different ages, genders, and education levels, although we may need to develop different screening criteria for people of different ages as aforementioned.

## 4. Discussion

The HK-GSDT is a new structural dialog task that can be used to screen for NCDs quickly. The test is brief (about 10 min) and easy to administer (can be administered by non-specialists with minimal training). Using a Hong Kong older adult sample in this study, we demonstrated that the test is reliable and valid for discriminating between different cognitive statuses (especially between M-NCDs and normal aging/m-NCDs). The HK-GSDT can be used as a standalone NCD screening test. It performed equally well with other established NCD screening tests, such as MoCA and HKLLT (and they all performed better than AD8). The HK-GSDT can also be used in conjunction with other established NCD screening tests to increase the accuracy of NCD screening—it explained additional variability in cognitive status on top of the other standardized tests (e.g., MoCA, HKLLT, AD8). In practice, clinicians often diagnose NCDs with multiple tests, which yield better classification accuracy than a single test [14].

A common limitation in these tests (HK-GSDT, MoCA, HKLLT, and AD8) gleaned from the current study was that none of them alone could discriminate m-NCD from normal aging well, although they were found to be effective in detecting m-NCDs or mild cognitive impairments in previous studies [45,50,53]. These findings could be due to several factors. It might be because these tests are all relatively easy for participants in both the normal-aging and the m-NCD conditions, thereby not sensitive enough to distinguish between them in the current study. It could also be because the sample size in our m-NCD condition was not big enough to reveal the discriminability of these tests. Another potential limitation of the HK-GSDT is that participants’ levels of culinary knowledge may influence their performance and processing/generation of speech [54]. In order to minimize the confounding effect of culinary knowledge, the HK-GSDT uses a dish that is familiar to almost every Chinese (in Hong Kong), so that all participants with normal cognitive ability should be able to recognize the ingredients. In addition, we do not score participants’ descriptions of cooking procedures, which are more dependent on culinary knowledge compared with recognizing dish ingredients. When using the HK-GSDT in other cultures, however, it is important to keep this potential limitation in mind. Users from other cultures may change the dish in the test to be more suitable for their cultures.

There are several potential solutions to approving the effectiveness of screening for NCDs (particularly m-NCDs). One potential solution is to increase the task difficulty in the future adaptations of the HK-GSDT, which may better distinguish between normal aging and m-NCDs. Another solution is to combine multiple tests to screen for m-NCDs [14]. Our results also showed that the conjunction of two tests yielded higher classification accuracy rates than a single test. Moreover, we could consider integrating the total test score and spoken language data (including text and audio data) from the HK-GSDT to screen for NCDs. A large body of studies has shown that language deficits occur at the very early stage of NCDs, and that using spoken language data (combined with modern machine learning and artificial intelligence techniques) can be highly accurate in detecting different kinds and stages of NCDs [26,27,28]. The analysis of spoken language is not the main focus of the current study, and we will develop a protocol for how to use the language data from the HK-GSDT to screen for NCDs in our follow-up studies.

Future studies of the HK-GSDT could take several directions. First, investigating whether it is possible to use the HK-GSDT to distinguish between different types of NCDs (e.g., AD, FTD, VD). We had a relatively small number of NCD participants and thus were not able to test this possibility in the current study. Second, administering the HK-GSDT to a larger sample that better represents the population in Hong Kong (or Chinese cultures), such that reliable cut-off points can be derived to distinguish between different cognitive conditions. Third, examining the applicability of the HK-GSDT in other cultures. Shopping is a daily activity common across cultures, and we expect the HK-GSDT task to be valid in different cultures. The dish and grocery products/layout in the task can be simply modified to fit other cultural contexts. Fourth, HK-GSDT can be used as an outcome measure for different types of intervention studies especially those directly targeting language and cognition [55,56,57,58]. Fifth, delineating the underlying neural mechanisms behind the various components of HK-GSDT via different modalities of task-free and task-related neuroimaging [59,60,61].

## 5. Conclusions

To conclude, the HK-GSDT is a brief, structural test that can be used, as a standalone test or in conjunction with other established tests, to screen for NCDs easily, efficiently, and effectively. We believe that the test can contribute to the screening for NCDs.

## Figures and Tables

**Table 1:** Cognitive functions involved in the Hong Kong grocery shopping dialog test (HK-GSDT).

Cognitive Domains	Specific Cognitive Functions
Perceptual-motor function	-Perceptual function: e.g., recognition of dish ingredients, perception of the store’s spatial layout. -Motor function: e.g., articulation of speech, speaking.
Attention and Processing speed	-Sustained attention: e.g., continual attention to the interconnected shopping task. -Selective attention: e.g., selective focus on the goal of purchasing the needed ingredients. -Processing speed: e.g., completing each section of the task within a certain timeframe.
Learning and Memory	-Semantic memory/knowledge: e.g., knowing the names of ingredients, knowing the membership relationships between ingredients and product categories (e.g., tomato belongs to vegetable). -Verbal episodic memory: e.g., the memory of (names of) ingredients and products. -Non-verbal episodic memory: e.g., the memory of visuospatial details, context, and locations. -Orientation: e.g., locating oneself in the grocery store.
Executive function	-Planning, focusing, monitoring, and directing behavior for the goal of getting needed ingredients. -Inhibition: e.g., inhibition of irrelevant information and products in the grocery store. -Switch: e.g., switching goal from purchasing one ingredient to purchasing another ingredient. -Working memory: e.g., holding the task goal in mind throughout the task.
Language	-Language comprehension: understanding what the tester says. -Language production: naming ingredients, naming actions, describing shopping steps. -Dialog speech between the tester and the participant.
Social cognition	[Social cognition is not highly involved in the test]

*Note.* The underlined cognitive functions are seldomly covered in the existing NCD screening tests.

**Table 2:** Sample demographics and diagnostic criteria for different cognitive conditions.

	M-NCD (Dementia)	m-NCD (Mild Cognitive Impairment)	Normal Aging	Total
Sample size (*N*)	42	39	464	545
Gender (% female)	56.1%	38.5%	60.8%	58.8%
Age (*M* ± *SD*)	81.8 ± 6.07	82.9 ± 4.37	71.8 ± 7.78	73.4 ± 8.37
Education (*M* ± *SD*)	6.1 ± 5.43	6.8 ± 5.09	8.4 ± 7.73	8.1 ± 7.44
Marital status (%)				
single/married/divorced/widow	0/61.9%/4.8%/33.3%	0/69.2%/10.3%/20.5%	7.3%/66.4%/6.5%/19.4%	6.2%/66.1%/6.6%/20.7%
Neurocognitive tests	usually AD8 ≥ 2 and HKLLT < −1.5 *SD* and MoCA ≤ 16th percentile	AD8 ≥ 2 &(HKLLT < −1 *SD* ORMoCA ≤ 16th percentile)	Scores out of the criteria for M- and m-NCD	
Clinical diagnosis	two clinicians	two clinicians	two clinicians	

*Note.* AD8 = Eight-item Interview to Differentiate Aging and Dementia; HKLLT = Hong Kong List Learning Test (2nd Edition); MoCA = Montreal Cognitive Assessment Hong Kong Version.

**Table 3:** Correlations between the HK-GSDT score and other measures (*N* = 545).

	*M*	*SD*	2	3	4	5	6	7	8	9	10	11	12	13	14
1. HK-GSDT	9.38	1.49	**−0.42**	−0.003	**0.12**	**−0.16**	**0.15**	**−0.16**	**0.42**	**0.29**	**0.35**	**0.35**	**0.56**	**0.58**	**0.60**
2. Age	73.37	8.37		0.08	**−0.11**	**0.18**	−0.07	**0.23**	**−0.42**	**−0.28**	**−0.41**	**−0.28**	**−0.45**	**−0.43**	**−0.40**
3. Gender	[58.8% female]				**0.12**	0.04	0.01	0.06	**−0.09**	0.01	0.04	**−0.14**	0.07	−0.01	−0.001
4. Education	8.14	7.44				−0.08	−0.002	−0.07	**0.10**	0.07	**0.12**	**0.11**	**0.12**	**0.19**	**0.13**
5. GDS	0.670	1.07					−0.04	**0.36**	**−0.20**	**−0.16**	**−0.20**	**−0.14**	**−0.22**	**−0.20**	**−0.22**
6. CCT	1.06	0.57						−0.03	0.04	0.01	0.01	0.02	0.05	0.05	0.01
7. AD8	3.26	2.36							**−0.20**	**−0.19**	**−0.18**	**−0.25**	**−0.30**	**−0.25**	**−0.25**
8. Logical memory	20.52	4.51								**0.32**	**0.42**	**0.47**	**0.50**	**0.52**	**0.51**
9. Digit span forward	8.12	1.34									**0.45**	**0.17**	**0.42**	**0.38**	**0.36**
10. Digit span backward	4.82	1.50										**0.33**	**0.49**	**0.52**	**0.44**
11. HKLLT	−0.45	0.90											**0.42**	**0.54**	**0.51**
12. mBNT	20.80	4.96												**0.72**	**0.59**
13. MoCA (full)	21.16	5.32													**0.74**
14. MoCA (5-min)	20.18	3.94													

*Note*. Significant (*p* < 0.05) correlations are marked in bold. HK-GSDT = Hong Kong Grocery Shopping Dialog Test; GDS = The Geriatric Depression Scale; CCT = Color Trails Test; AD8 = Eight-item Interview to Differentiate Aging and Dementia; HKLLT = Hong Kong List Learning Test (2nd Edition); mBNT = modified Boston Naming Test (30 items); MoCA = Montreal Cognitive Assessment (the Hong Kong version), including both the full version and the 5 min short version.

## Data Availability

Data used in the current study are available upon request.

## Supplementary Materials

The following supporting information can be downloaded at: https://www.mdpi.com/article/10.3390/ijerph192013302/s1, Figure S1: images used in the Hong Kong Grocery Shopping Dialog Task (HK-GSDT); Figure S2: Factor structure of the five items in the Hong Kong Grocery Shopping Dialog Task (HK-GSDT); Table S1: areas under the ROC curves (and 95% confidence intervals) for different tests to discriminate between various cognitive health conditions; Table S2: The goodness-of-fit indices of the multinomial logistic regression models that predict cognitive health conditions.
