# Supplementary material for: The Hong Kong Grocery Shopping Dialog Task (HK-GSDT): A Quick Screening Test for Neurocognitive Disorders
Source: Int J Environ Res Public Health. 2022 Oct 15;19(20):13302. doi: 10.3390/ijerph192013302 (PMC9603616; doi:10.3390/ijerph192013302)
Supplement: Supplementary file 1 [file ijerph-19-13302-s001.zip › ijerph-1949129-supplementary.pdf]

The Hong Kong Grocery Shopping Dialog Task (HK-GSDT):
A Quick Screening Test for NCDs

Supplementary Materials

1. Supplementary Tables

Table S1. Areas under the ROC curves (and 95% confidence intervals) for different tests to discriminate between various cognitive health conditions

	HK-GSDT	5-min MoCA	full version MoCA	HKLLT	AD8
M-NCD vs. m-NCD and normal aging	.89***	.91***	.89***	.92***	.62*
	[.83, .96]	[.87, .95]	[.84, .94]	[.87, .97]	[.52, .72]
M-NCD vs. m-NCD	.81***	.88***	.85***	.90***	.41
	[.72, .91]	[.80, .95]	[.76, .93]	[.82, .98]	[.29, .54]
m-NCD vs. normal aging	.67**	.57	.61*	.69**	.74***
	[.57, .77]	[.48, .67]	[.52, .70]	[.60, .77]	[.65, .82]
normal aging vs. m-/M-NCD	.79***	.75***	.76***	.81***	.68***
	[.72, .85]	[.68, .81]	[.70, .82]	[.75, .86]	[.62, .75]

Note. * Asymptotic significance $p < .05$, ** $p < .01$, *** $p < .001$; a larger area under the ROC curve indicates a better performance on discriminating between cognitive conditions. HK-GSDT = Hong Kong Grocery Shopping Dialog Test; MoCA = Montreal Cognitive Assessment Hong Kong Version; HKLLT = Hong Kong List Learning Test (2nd Edition); AD8 = Eight-item Interview to Differentiate Aging and Dementia.

Table S2. The goodness-of-fit indices of the multinomial logistic regression models that predict cognitive health conditions

Regression models $	Goodness-of-fit indices				Model comparison #
	AIC	*BIC*	-2 log-likelihood	pseudo R^{2*}	
Model 1: Demographics (D.)	386.88	421.20	370.88	.35	Model 2 vs. Model 1:
Model 2: D. + HK-GSDT	375.23	418.11	355.23	.47	$\chi^2 = 56.15$, *df* = 2, *p* < .001
Model 3: D. + MoCA (5-min)	375.94	418.84	355.94	.49	Model 4 vs. Model 3:
Model 4: D. + MoCA (5-min) + HK-GSDT	365.96	417.42	341.96	.52	$\chi^2 = 12.86$, *df* = 2, *p* = .002
Model 5: D. + MoCA (full)	383.62	426.37	363.62	.46	Model 6 vs. Model 5:
Model 6: D. + MoCA (full) + HK-GSDT	372.84	424.14	348.84	.50	$\chi^2 = 18.94$, *df* = 2, *p* < .001
Model 7: D. + HKLLT	356.36	399.24	336.36	.53	Model 8 vs. Model 7:
Model 8: D. + HKLLT + HK-GSDT	339.94	391.40	315.94	.57	$\chi^2 = 23.19$, *df* = 2, *p* < .001
Model 9: D. + AD8	428.50	471.39	408.50	.37	Model 10 vs. Model 9:
Model 10: D. + AD8 + HK-GSDT	379.81	431.26	355.81	.49	$\chi^2 = 56.82$, *df* = 2, *p* < .001

Note. $ The outcome variable in these models was cognitive health condition as a categorical variable (normal aging, m-NCD, or M-NCD); * pseudo R^2 was Nagelkerke R^2; # model comparison was conducted using likelihood ratio test; HK-GSDT = Hong Kong Grocery Shopping Dialog Test; MoCA = Montreal Cognitive Assessment Hong Kong Version; HKLLT = Hong Kong List Learning Test (2nd Edition); AD8 = Eight-item Interview to Differentiate Aging and Dementia.

2. Supplementary Figures

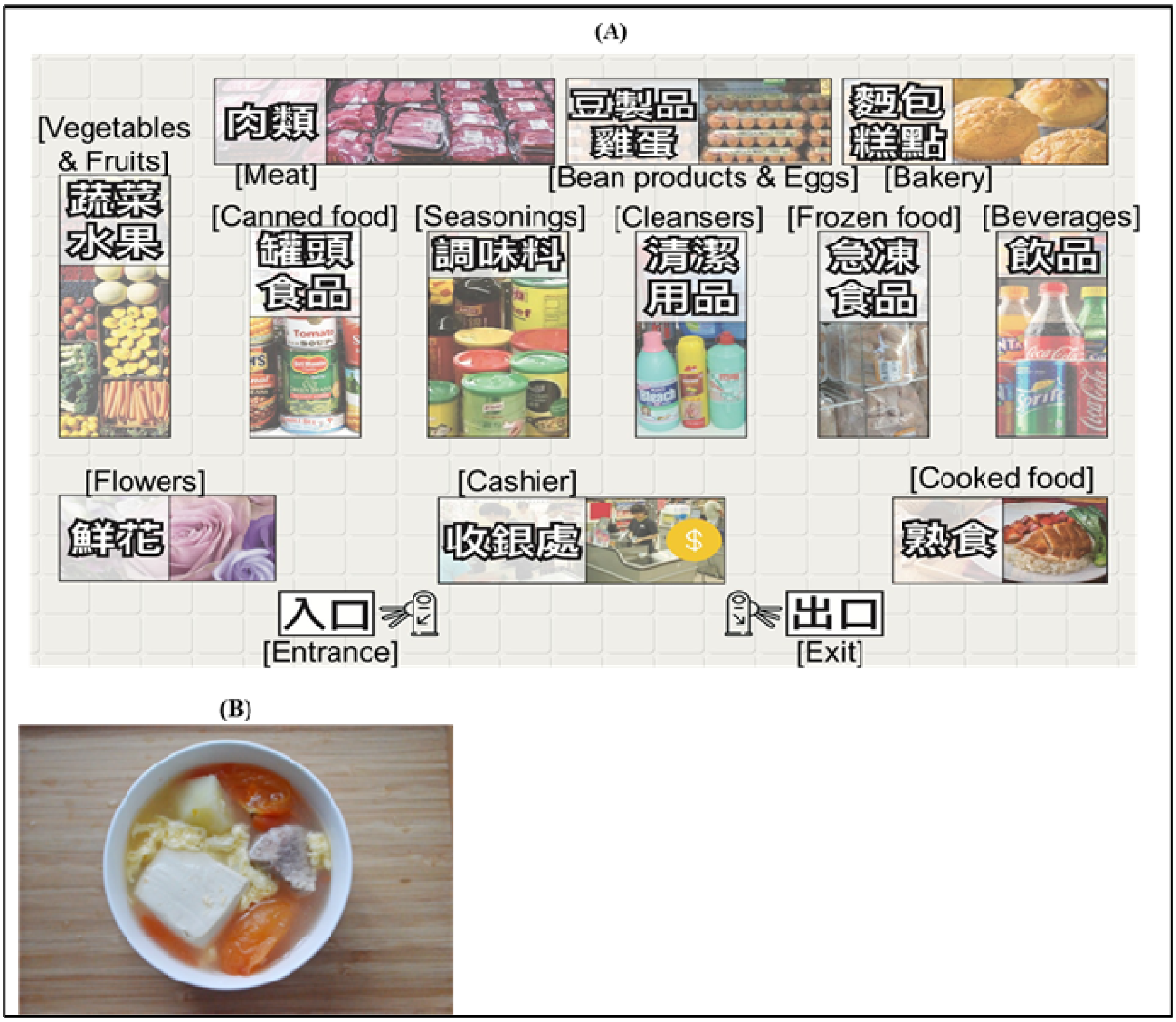

Figure S1. Images used in the Hong Kong Grocery Shopping Dialog Task (HK-GSDT). Panel (A) displays the layout of a hypothetical grocery store. Panel (B) is the picture of a traditional Chinese soup, which is made with tomatoes, tofu, eggs, pork, white radishes, salt, and water.

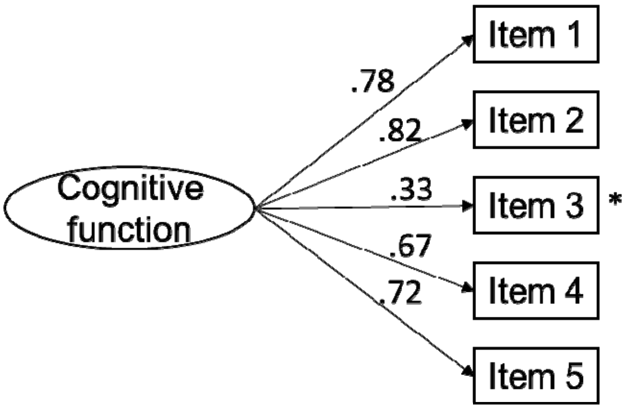

Figure S2. Factor structure of the five items in the Hong Kong Grocery Shopping Dialog Task (HK-GSDT). * The score on Item 3 was reverse coded. The numbers on the arrows are factor loadings.
